# Dendritic Cells and* Leishmania* Infection: Adding Layers of Complexity to a Complex Disease

**DOI:** 10.1155/2016/3967436

**Published:** 2016-01-19

**Authors:** Daniel Feijó, Rafael Tibúrcio, Mariana Ampuero, Cláudia Brodskyn, Natalia Tavares

**Affiliations:** ^1^Centro de Pesquisas Gonçalo Moniz (CPqGM), 40296-710 Salvador, BA, Brazil; ^2^Universidade Federal da Bahia (UFBA), 40170-115 Salvador, BA, Brazil; ^3^Instituto de Investigação em Imunologia (iii), 01246-903 São Paulo, SP, Brazil

## Abstract

Leishmaniasis is a group of neglected diseases whose clinical manifestations depend on factors from the host and the pathogen. It is an important public health problem worldwide caused by the protozoan parasite from the* Leishmania* genus. Cutaneous Leishmaniasis (CL) is the most frequent form of this disease transmitted by the bite of an infected sandfly into the host skin. The parasites can be uptook and/or recognized by macrophages, neutrophils, and/or dendritic cells (DCs). Initially, DCs were described to play a protective role in activating the immune response against* Leishmania* parasites. However, several reports showed a dichotomic role of DCs in modulating the host immune response to susceptibility or resistance in CL. In this review, we discuss (1) the interactions between DCs and parasites from different species of* Leishmania* and (2) the crosstalk of DCs and other cells during CL infection. The complexity of these interactions profoundly affects the adaptive immune response and, consequently, the disease outcome, especially from* Leishmania* species of the New World.

## 1. Introduction

Leishmaniasis are a complex of vector-borne diseases caused by an intracellular protozoan parasite from* Leishmania* sp. (Kinetoplastida, Trypanosomatidae). Its clinical spectra depends largely on parasite species and host immune response. Although the disease has been known and studied for a long time, it is still considered as a neglected and public health problem worldwide. Such diseases affect approximately 12 million people in 88 countries, where 350 million inhabitants are exposed, mainly in remote rural areas and underserved urban areas [1]. The clinical forms range from asymptomatic infection to two main clinical syndromes: visceral leishmaniasis (VL) and cutaneous leishmaniasis (CL).

VL is a chronic infection, fatal if not treated. It is characterized by progressive fever, weight loss, splenomegaly, hepatomegaly, anemia, and spontaneous bleeding associated with marked inflammatory imbalance [2]. The hallmark of this disease is thought to be a lack of cellular immune response against the parasite and high systemic levels of IFN-g and IL-10 [3].

CL is the most frequent form of this disease. It is characterized by chronic evolution, which affects the skin and cartilaginous structures [4]. The main clinical forms of diseases associated with CL are the Localized Cutaneous Leishmaniasis (LCL), Mucocutaneous Leishmaniasis (ML), disseminated and diffuse Leishmaniasis [1].

LCL is mainly caused by the species* Leishmania tropica*,* L. aethiopica,* and* L. major* in the Old World. However, New World LCL is mainly caused by multiple species of both* Leishmania* subgenera* Leishmania* (*L. amazonensis*,* L. infantum*,* L. mexicana, and L. venezuelensis*) and* Viannia* subgenera (*L. braziliensis*,* L. guyanensis*,* L. panamensis*, and* L. peruviana*). The incubation period lasts on average from 2 weeks to 3 months with the appearance of papules or nodules and, sometimes, is preceded or accompanied by the swelling of underlying nodes. The hallmark of this illness is the development of single or multiple ulcerated dermal lesions. Over time, the lesion may evolve spontaneously to healing or develop into different frames of gravity in ulceration of the lesion with its expansion [4].

Some patients (a fraction of 3%) may develop the ML, caused by the infection with* L. braziliensis* and* L. guyanensis*. The symptoms are associated with the destruction of the nasal cavity and oropharyngeal tissues [4]. Genetic diversity of* Leishmania* species contributes to the difficulty of controlling the disease and to the increase in the number of cases that are resistant to conventional treatment [5]. Although both forms of CL are rarely fatal, they can cause nasty scars on the skin and severe problems in the oropharyngeal device [4].

Dendritic cells (DCs) are a family of professional antigen-presenting cells (APCs) that resides in all peripheral tissues in an immature state, capable of antigen uptake and processing. As such, they function as sentinel of the immune system. After contact with microorganisms or substances associated with infection or inflammation, DCs undergo a process of maturation and migrate to the T cell areas of lymphoid organs. There, they present antigens to naïve T cells and modulate their responses [6]. The maturation process consists of (1) increased expression of major histocompatibility complex (MHC) and costimulatory molecules, such as CD40, CD80, CD86, and CD54; (2) downregulation of antigen capture and phagocytic capacity; (3) enhanced cytokine secretion; (4) different patterns of chemokine receptor expression and chemokine production, enabling DC migration and recruitment of other cell types [7, 8].

DCs are able to take up antigens via different groups of receptor families, such as Fc receptors, C-type lectin receptors (CLRs), and pattern recognition receptors (PRRs), such as Toll-like receptors (TLRs) [9]. The engagement between ligand and its receptor enables DCs to recognize a wide range of microbial stimuli [10].

DCs are a heterogeneous population of cells that can be divided into 2 main categories: the plasmacytoid DCs (pDCs), experts in type I interferon synthesis, and the conventional DCs (cDCs), specialized in antigen capture, processing, and presentation for T cell priming. pDCs constitutively express MHC class II molecules and lineage markers, such as CD45RA/B220^+^, Ly6C/GR-1^+^, and siglec-H [11–13]. Two cDCs subsets can be distinguished based on functional specialization. cDC1s are particularly efficient in CD8^+^ T cell activation and cross-presentation. cDC2s are most efficient for CD4^+^ T helper polarization, especially Th2 or Th17 [14]. In mice, cDC1s express high levels of CD8*α* or CD103 [15, 16] and cDC2s express CD11b and CD172a (also known as SIRP*α*) [17]. In humans, DCs can be subdivided into two main populations: CD141^+^ DCs (also referred to as BDCA3^+^) and CD1c^+^ DCs (also known as BDCA1^+^). Based on gene expression profiles and functions similarities, human CD141^+^ DCs and CD1c^+^ DCs resemble those of mouse cDC1s and cDC2s, respectively [18–21]. Also, monocytes can adopt a DC morphology and antigen-presenting functions in inflammatory sites, leading to their designation as monocytes-derived DCs (MoDCs) [22, 23]. In mice, MoDCs derived from Ly6C^hi^ monocytes can express CD11c and MHC class II, and, similarly to macrophages, F4/80 and CD64 [23, 24]. In humans, MoDCs derived from CD14^+^ monocytes and can express CD1a [24]. Langerhans cells (LCs) present DC morphology and antigen-presenting functions in the skin [25, 26]. They constitutively express major histocompatibility complex (MHC) class II and high levels of the lectin Langerin [27]. The most current phenotypes described for each type of DC are summarized in Table 1.

Several reports show a central role for DCs in orchestrating immune responses in leishmaniasis [28–30]. In this review, we discuss the heterogeneity of the interaction between DCs and different species of* Leishmania* that causes CL.

## 2. Interaction of DC with Different* Leishmania* Species

Infection with* Leishmania* parasites leads to lifelong immunity against the same subspecies, after the infection is healed. Experimental models of CL infections are largely used to study the mechanism under this lifelong immunity. Most of these studies have been carried out by inoculation of* L. major*, a species present in the Old World. However, experimental studies with the New World* Leishmania* sp., such as* L. amazonensis* and* L. braziliensis*, are scarce. This reinforces the importance of studies about the immune response induced by specific species of* Leishmania*.

### 2.1. Interaction of DC Subtypes with* Leishmania major*

Current paradigms of the involvement of T helper subsets in infectious diseases are based, in large part, on the results of studies about resistance and susceptibility to* L. major* in inbred mice. In murine LCL, BALB/c mice respond to infection with production of Th2-type cytokines, in particular IL-4 and IL-10. These cytokines are associated with disease progression and susceptibility to* L. major*. In contrast, recovery from infection of resistant mice (e.g., C57BL/6) depends on the induction of a polarized Th1-type response, resulting in macrophage activation and killing of parasites.

Early studies demonstrated that epidermal LCs phagocyte* L. major in vivo* and migrate to draining lymph nodes (dLNs) for presentation to antigen-specific T cells [31]. However, later studies showed that DCs harboring parasites in dLNs are Langerin negative and express dermal DC markers [32]. Besides, mice deficient for MHC class II exclusively in LCs (but not in dermal DCs) control* L. major* infection, similar to wild type animals [33]. This finding suggests that LCs are dispensable for triggering T cell response during* Leishmania* infection. Moreover, a recent study showed that LCs might even play a pathogenic role during low dose infection via the induction and expansion of regulatory T cells [34]. Some studies showed that dermal DCs harboring parasites migrate out of the skin and transport antigens to the dLNs [32, 35]. Another study suggested that blood MoDCs might phagocyte parasites and transport them to the dLN, where they present parasite-derived antigen to T cells [29]. In this way, depending on the tissue and the subtype involved, DCs could have different biological response towards* Leishmania* interaction.

The production of IL-12 by APCs is critically important for the polarization of naïve T cells toward Th1 subset and subsequent IFN-*γ* production [30, 36]. Infection of DC with* L. major* results in functional IL-12p70 production [37]. Interestingly, DC subsets are differentially permissive to* Leishmania* parasites and this differential infectivity seems to be inversely correlated with the ability of infected cells to produce IL-12p70 [38, 39]. CD8*α* ^+^ DCs are less permissive to* L. major* amastigotes compared to CD8*α* ^−^ DCs. However, CD8*α* ^+^ and CD103^+^ DCs are the most powerful IL-12p70 producers in response to this infection [36, 38]. The mechanism(s) that control the induction of IL-12 from DCs and the functional differences between IL-12-producing DCs and nonproducers are still not known.

It has been speculated that different outcomes of* Leishmania* infection between resistant and susceptible mice may be related to differences in their DC functions, particularly in the differentiation of naïve TCD4^+^ into effector cells [40, 41]. However,* L. major*-infected skin-derived DCs from BALB/c and C57BL/6 mice upregulated costimulatory molecules and produced comparable levels of proinflammatory cytokines [30]. In further contrast, LCs from BALB/c mice upregulate IL-4 receptor expression and downregulate IL-12p40 production in response to* L. major* infection [42]. These findings suggest that* L. major* is able to inhibit Th1 immune response through altering DCs functions, depending on the cell type involved. Baldwin et al. [43] found that* L. major*-infected BALB/c mice have an increased number of plasmacytoid DCs in their dLNs [43]. This was associated with increased pDC recruitment early after infection, compared to infected C57BL/6 mice.

Ashok and Acha-Orbea [44] proposed a model of infection based on DCs subtypes at the different time points after* L. major* infection. This model nicely explains many features and contradiction in the role of DCs subsets in cutaneous leishmaniasis: (1) dermal DCs and LCs play a role early in infection and (2) monocyte-derived dendritic cells and lymph node resident DCs are important to establish an efficient immune response at later time points [44]. However, this proposed model only focuses on DCs role in murine models based on* L. major* infection. It is not clear whether the differences observed in DCs from susceptible and resistant mice are relevant to the pathogenesis of the disease in humans. At present, there is still limited information on initial or late DC responses to other species of* Leishmania* and their contribution to prime protective or pathogenic T cell responses in cutaneous leishmaniasis.

### 2.2. Role of DCs Interaction with Other* Leishmania* Species

Even though cutaneous leishmaniasis is caused by almost 20 species of* Leishmania*, most studies about the role of DCs are focused on experimental models of 4 species:* L. major*,* L. mexicana*,* L. amazonensis, and L. braziliensis*.

The role of Langerhans cells (LCs) was examined in patients with different forms of cutaneous leishmaniasis (CL) caused by the New World* Leishmania* sp. (*L*.* braziliensis*,* L*.* mexicana*,* and L*.* amazonensis*) [45, 46]. The analysis of LCs density among different clinical forms of CL showed a reduced LC density in* L. braziliensis* infection with a positive DTH response (delayed type IV hypersensibility). In comparison to nonreactive DTH from severe forms caused by* L. amazonensis*, an increase of LC density was observed [46]. These results indicate a species-specific negative correlation between LC density and DTH reaction among clinical forms of CL. This could lead to a suppression of T cell immune response. However, in CL caused by* L. mexicana*, the LCs density is similar between mild and severe clinical forms [45]. These findings indicate that* L. amazonensis* may use LCs to prime regulatory T cells, inhibiting the T cell responses, in a similar way to* L. major* infection [34].

Moreover, corroborating this clinical observation, experimental evidence confirms that early stages of* L. amazonensis* infection in BALB/c mice may impair multiple immune functions, leading to an antigen-specific T cell immune suppression [47]. Similar results were observed in murine and human DCs infected* in vitro* by* L. amazonensis* [48, 49]. However, for* L. braziliensis* murine infection, a full DC maturation process and activation were observed [50]. Together, these studies point out the specificity of strategies from different* Leishmania* species to modulate T cell immune response through DCs. Besides, there is a lack of information about the importance of other DC types for the development of different clinical forms caused by one species.

The dynamics of DCs migration to lymph nodes and to nonlymphoid tissues is also an important issue for the disease outcome. DCs progenitors and monocytes terminally differentiate into DCs subsets, depending on the nonlymphoid tissue they migrate, such as the skin. When activated, skin DCs upregulate CCR7 and migrate again to draining lymph node via afferent lymphatics in response to CCL19 and CCL21 [14, 51]. The migration of monocyte-derived DCs to the lymph nodes is driven by CCR2 and its ligands [52]. In VL, there is a lack of protective immune response, partially, due to an altered DC migration to the spleen and dLNs [53–56]. This is also observed in CL. During* L. major* infection, MoDCs are preferentially recruited to the infected skin and dLN. They are important to mediate a Th1 response and to control the infection [29]. Such enhanced recruitment of DCs to dLN leads to hypertrophy of the LN, which is associated with a protective response against* L. major* [57]. On the other hand,* L. mexicana* infection induces limited recruitment of MoDCs and decreased LN expansion, without affecting T cell proliferation [58, 59]. This diminished recruitment is independent of IL-10 and leads to disease progression, since treatment with neutralizing antibodies against IL-10 increases MoDCs migration and decreases parasite burden [59]. The modulation of DC recruitment to the infected skin and dLN could be used as a mechanism of immune evasion by different* Leishmania* sp. that causes CL.

## 3. Differences in Recognition of* Leishmania* Parasites by DCs

DCs express a wide variety of pattern recognition receptors (PRRs) that are important for initiating and directing subsequent adaptive immunity. The recognition of pathogen associated molecular patterns (PAMPs) can vary among species of* Leishmania*. de Veer et al. [60] found that MyD88 deficient (MyD88^−/−^) C57BL/6 mice are more susceptible to* L. major* infection, suggesting a critical role of TLR signaling in initiating anti-*Leishmania* immunity [60]. They further demonstrated that LPG, the most abundant surface molecule of* Leishmania* and a TLR2 ligand, is responsible for the generation of protective immunity against leishmaniasis. Neutralization of TLR2 and TLR4* in vivo* reduced the expression of costimulatory molecules on DCs infected with* L. major* [61]. However, the lack of TLR2 in mice infected with* L. braziliensis* resulted in an enhanced DC activation and increased IL-12 production. As such,* L. braziliensis*-infected DCs from TLR2^−/−^ were more competent in priming naïve CD4^+^ T cells* in vitro*. These findings correlated with an increased IFN-*γ* production* in vivo* and enhanced resistance to infection [62]. On the other hand,* L. braziliensis*-infected DCs from MyD88^−/−^ exhibited less activation and decreased production of interleukin-12 [62].

Furthermore, it has been shown that TLR9 signaling is crucial to the release of IL-12 and type I IFN from DCs exposed* in vitro* to* L. major* and* L. braziliensis*.* In vivo* assays with* L. major* infection also confirmed the importance of TLR9 to IL-12 production from DCs [63, 64]. However, for* L. braziliensis* infection,* in vivo* experiments established that TLR9^−/−^ mice could generate a Th1 response and activate DC, despite the diminished DC activation* in vitro* [65]. Together these data from TLR assays reinforce the importance to define* in vitro* and* in vivo* approaches to better characterize the modulation of DC induced by different* Leishmania* sp. on the immune response.

The recognition of pathogens could be optimized by the action of antibodies, a process called opsonization [66]. The uptake of* L. amazonensis* amastigotes by DC and LCs can be promoted by opsonization [67]. This process leads to IL-10 production from these DCs, as well as the consequent priming of IL-10-producing T CD4^+^ and lesion progress in mice [67]. In contrast, the uptake of opsonized* L. major* by murine DCs leads to cell activation, IL-12 production, and protective immunity [68, 69]. In further contrast,* L. mexicana* and* L. braziliensis* are highly efficient in infecting DCs, even in the absence of antibodies [62, 70]. These findings point out that the profile of cytokine production from DC is differently induced in a species-specific way, despite the same pathway recognition of* Leishmania*.

## 4. Interaction of DC with Other Leukocytes

Dendritic cells are the most important APCs, making a link among innate and adaptive immunity. They can have direct and diverse functions on the immune response, leading to activation as well as tolerance and anergy. In the context of CL, the functions of DCs could be modulated by the interaction with other leukocytes, such as neutrophils and NK cells.

It has been shown that genomic DNA of* L. major* and* L. braziliensis* promastigotes activate cDCs and pDCs to produce IL-12 and IFN-*α*/*β*, respectively. After, they were cocultured with NK cells, leading to an increased IFN-*γ* release and NK cytotoxicity [63, 64]. Certain* Leishmania* species (*L*.* tropica*,* L. amazonensis*, and* L. mexicana*), in their amastigotes phase, are poor inducers of IL-12 by DC. This might account for the limited NK cell response during prolonged infections* in vivo* [71, 72]. Hernandez Sanabria et al. [73] demonstrated that infection of* L. amazonensis* amastigotes triggers minimal DC activation, but the interaction with activated NK cells could partially overcome the deficiencies in DC activation* in vitro* [73]. The injection of activated NK cells 24 hours after infection* in vivo* promoted IL-12 release and increased the expression of costimulatory molecules in infected DCs (CD40, CD83, and CD80) [73]. Regarding NK cells in this context, they showed increased expression of IFN-*γ* and CXCL10. Such interaction forms a positive loop, leading to the induction of a Th1 immune response to reduce parasite loads.

In a vaccination context against* L. major*, BALB/c depleted of NK cells and vaccinated with DCs pulsed with parasites lysates and, then, challenged with* L. major* showed a significant increase in footpad swelling and parasite load in the dLN [74]. In order to evaluate the mechanisms under this process, coculture of these cells was assessed. This resulted in upregulation of CD69 and IFN-*γ* on NK cells as well as CD86 and MHC-II on pulsed DCs. The interaction of DC and NK cells is a good example of a positive interaction that leads to cross activation and host immune protection, either, in the context of an infection or vaccination.

**Figure 1:**
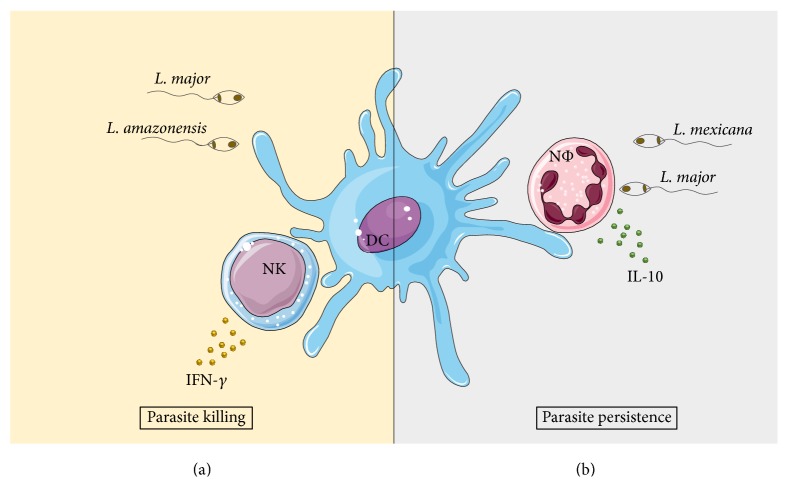
Interaction of DC with different leukocytes in the cutaneous leishmaniasis context. (a) shows the interaction among NK and infected DCs that leads to host immune protection and parasite killing through IFN-*γ* production during* L. amazonensis* [73] or* L. major* [74] infection. (b) shows the outcome induced by the increased production of IL-10 after the interaction between infected neutrophils (NΦ) with* L. major* [75] or* L. mexicana* [76] and DCs, leading to parasite persistence.

Neutrophils are also an important cell type which interact with DCs. The ingestion of* L. major* by neutrophils in parasite-inoculated mice increased cell apoptosis. This favored the capture of apoptotic neutrophils by DCs, preventing the activation of infected DCs in the skin [75]. In the case of* L. mexicana* infection, this effect was not observed, since the infection did not induce neutrophil apoptosis. Parasites sequestration by neutrophils impaired DC migration to the site of infection, through reduced CCL2, CCL3, and CCL5 release. Furthermore, the diminished DCs that migrate to the site of infection had a decreased motility and parasite uptake [76]. The interaction among DCs and neutrophils is a good example of negative regulation of the immune response, regardless of the* Leishmania* sp. Although the consequences of DCs and neutrophils interaction lead to immune suppression, the mechanisms of action could be diverse and* Leishmania* species-specific (summarized in Figure 1).

## 5. Systems Biology as a Tool to Develop Vaccines against CL Based on DCs

Recently, Matos et al. [77] showed the potential of targeting DC* in vivo* for induction of protective immune response against* L. major* in murine model of CL [77]. However, the role of DCs in CL is diverse and complex. Such role could explain some unique clinical manifestations, depending on the species of* Leishmania* that causes the disease. Because of that, the use of vaccines based on DCs is not yet a reality for CL. This is not only due to the complexity of the disease. Genomic and transcriptional profiles vary not only interspecifically in* Leishmania* parasites that causes CL [78–81], but also within parasites strains isolated from patients [82]. This variability leads to a difficult task to identify a universal antigen vaccine candidate to clinical trials.

Systems Biology could be a useful tool to overcome these difficulties. Systems Biology have a holistic approach to describe complex interactions between multiple components in a biological context [83]. Using high dimensional molecular approaches, Systems Biology identifies changes caused by perturbations, such as infection or vaccination, combined with computational analysis to model and predict responses [84]. The first studies of Systems Biology about the immune response predicted that certain signatures of CD8^+^ T cells and B lymphocytes correlated with a protective immune response induced by a vaccine against Yellow Fever Virus [85, 86]. Since then, there is an increased interest about the research of immune responses based on Systems Biology approaches. These studies lead to the identification of interactions between pathogens and hosts and factors for parasite dissemination and disease progression, as well as to the selection of promising antigens as vaccine candidates [87–89]. For instance, hub genes with unknown functions were identified from* Plasmodium falciparum* parasites isolated from noncerebral clinical complications of malaria. The presence of these genes correlates parasite burden and survival with complicated clinical manifestations [90]. These findings revealed the crucial roles of these genes in parasite biology and their potential as candidates for intervention strategies.

Regarding leishmaniasis, Albergante et al. [91] have developed an* in silico* Petri net model that simulates hepatic granuloma development during the infection in experimental visceral context. This model identified an intergranuloma diversity of the antileishmanial activity and a dominant regulatory role of IL-10 produced by infected Kupffer cells at the core of the granuloma [91]. This approach raised new insights into how effector mechanisms may be regulated within the granuloma and revealed a useful tool to interpret how interventions may operate. For cutaneous leishmaniasis, the analysis of DNA sequence of* L. braziliensis* and* L. guyanensis* isolated from patients with different treatment outcomes identified polymorphisms related to drug resistance [92]. This study showed that genes related to drug resistance could be used to discriminate the two species of the subgenus* L. Viannia* and also could predict treatment failure.

Those studies mentioned above demonstrate the use of System Biology as a useful tool to better understand an infection, to identify unknown pathogen cell signaling pathways, potential biomarkers of disease susceptibility, and immunological alterations that aggravates the pathology. However, the studies employing this approach are few, but they will be very promising for the development of new technologies on the leishmaniasis field.

A successful application of the System Biology approach was modeled to study the function and the role of pDC during cytopathic virus infection to identify multiscale interactions involved in the protection against the virus [93]. The results obtained from this analysis identified and predicted that (1) one infected pDC secretes sufficient type I IFN to protect up to 10^4^ macrophages from cytopathic viral infection; (2) pDC population in the spleen protects against virus variants which inhibit IFN production; and (3) antiviral therapy should primarily limit viral replication within peripheral organs. Together, these results demonstrate the importance of System Biology application to direct and optimize the use of different technologies based on DCs.

In this way, the application of System Biology could be a useful tool to design and develop promising vaccines candidates based on DCs pulsed with* Leishmania* antigens. Studies about DC signaling network based on Systems Biology approach are already published and they stand for the feasibility of this technique [94, 95]. However, the development of vaccines based on DCs through Systems Biology approach needs to be well designed to avoid undesired effects, such as the exacerbation of the CL through the increase of inflammation [96].

## 6. Conclusion and Future Directions

Given the fact that the disease pathology of CL is highly variable depending on the species of* Leishmania*, it is very hard to generalize specific modulatory mechanisms to all strains and in all hosts. This is important because most of the studies about the role of DCs during* Leishmania* infection were usually conducted with a single species of the parasite, which precludes multi-species/strain comparison. Not all* Leishmania* species and its interaction with DCs were studied. For instance, infection caused by* L. guyanensis* paradoxically induces a specific immune response via TLR3 early after infection that impairs killing of parasites [97]. A more comprehensive study would be very helpful for a better understanding about the role of these cells and the mechanisms that regulate their antigen presentation functions and also pathogen factors that could influence the antigen presentation and subsequent activation of the adaptive immune system. Besides that, the development and use of computational immunology have been constantly increasing its value. Nowadays, different* in silico* approaches are available for identification of potential epitopes and antigens for vaccines, since experimental methods are difficult and time-consuming [98]. In addition, the DNA sequencing techniques became less expensive and, therefore, many parasite genome strains can be sequenced. Their predicted proteomes can be assessed considering their variability, an important feature of antigen candidates for vaccine development to one or all* Leishmania* species that cause CL. In this way, the use of DCs is promising for generation of potential alternatives therapies and vaccines protocols to improve the quality of life of patients infected by these protozoan parasites.

## Figures and Tables

**Table 1:** Summary of current phenotypes described for different DC subsets.

DC type	Phenotype/markers	Function	Reference
Plasmacytoid DC (pDC)	MHC-II, CD45RA/B220, Ly6C/GR-1, Siglec-H	Type I-IFN synthesis	[11–13]
Conventional DC type 1 (cDC1)	CD8*α*, CD103 (mice); CD141/BDCA3 (humans)	Antigen cross-presentation, CD8*α* ^+^ T cell activation	[15, 16, 18, 19]
Conventional DC type 2 (cDC2)	CD11b, CD172/SIRP*α* (mice); CD1c/BDCA1 (humans)	CD4^+^ T cell polarization	[17, 20, 21]
Monocyte-derived DC (MoDC)	CD11c, MHC-II, F4/80, CD64 (mice); CD1a (humans)	Antigen presentation at inflammatory sites	[23, 24]
Langerhans cell (LC)	MHC-II, Langerin	Antigen presentation in the skin	[25–27]
