# Corrigendum to “Dendritic Cells and *Leishmania* Infection: Adding Layers of Complexity to a Complex Disease”

**DOI:** 10.1155/2018/1236737

**Published:** 2018-03-22

**Authors:** Daniel Feijó, Rafael Tibúrcio, Mariana Ampuero, Cláudia Brodskyn, Natalia Tavares

**Affiliations:** ^1^Centro de Pesquisas Gonçalo Moniz (CPqGM), 40296-710 Salvador, BA, Brazil; ^2^Universidade Federal da Bahia (UFBA), 40170-115 Salvador, BA, Brazil; ^3^Instituto de Investigação em Imunologia (iii), 01246-903 São Paulo, SP, Brazil

The article titled “Dendritic Cells and *Leishmania* Infection: Adding Layers of Complexity to a Complex Disease” [1] was found to contain material from published work without citation, as follows:

- Dong Liu and Jude E. Uzonna, “Macrophages and dendritic cells and its influence on the host immune response,” *Frontiers in Cellular and Infection Microbiology*, 2:83, 2012:
  - In the paragraph beginning with “Several reports show…”
  - In the paragraphs beginning with “Early studies demonstrated…where they present parasite-derived antigen to T cells,” “The production of IL-12 by APCs … downregulate IL-12p40 production in response to *L. major* infection,” “Baldwin et al. found…infected C57BL/6 mice,” and “It is not clear whether…protective or pathogenic T cell responses.”
  - In the paragraph beginning with “DCs express a wide variety…immunity against *leishmaniasis*.”
  - In the paragraphs beginning with “Given the fact that…multi-species/strain comparison” and “A more comprehensive…activation of the adaptive immune system.”
- Olga Brandonisio, Rosa Spinelli, and Maria Pepe: “Dendritic cells in Leishmania infection,” *Microbes and Infection*, vol. 6, Issue 15, pp. 1402–1409, December 2004, http://www.sciencedirect.com/science/article/pii/S1286457904003053:
  - In the paragraph beginning with “Dendritic cells (DCs) are a family of professional antigen-presenting cells (APCs)…a wide range of microbial stimuli.”
- Von Stebut, “Cutaneous *Leishmania* infection: progress in pathogenesis research and experimental therapy,” *Experimental Dermatology*, 16: 340–346, 2007, http://onlinelibrary.wiley.com/doi/10.1111/j.1600-0625.2007.00554.x/abstract:
  - In the sentence beginning with “Infection with *Leishmania* parasites…after the infection is healed” and in the paragraph beginning with “Current paradigms…activation and killing of parasites.”
- Rafael de Freitas e Silva, Maria Carolina Accioly Brelaz de Castro, Antônio Mauro Rezende, and Valéria Rêgo Alves Pereira, “Targeting dendritic cells as a good alternative to combat *Leishmania* spp.,” *Frontiers in Immunology*, 26 November 2014, https://doi.org/10.3389/fimmu.2014.00604:
  - In the paragraph beginning with “Nowadays, different in silico approaches…one or all *Leishmania* species that cause CL.”

The first author Daniel Feijó apologizes for these errors.
